# Linguistic Analysis of Online Domestic Violence Testimonies in the Context of COVID-19

**DOI:** 10.1177/21582440221146135

**Published:** 2023-01-10

**Authors:** Valentin L. Buchner, Sharina Hamm, Barbara Medenica, Marc L. Molendijk

**Affiliations:** 1 Leiden University, The Netherlands; 2 Leiden University Medical Center, The Netherlands

**Keywords:** COVID-19, domestic violence, linguistic analysis, Reddit, mental health, social support, leisure

## Abstract

Worldwide, an increase in cases and severity of domestic violence (DV) has been reported as a result of social distancing measures implemented to decrease the spreading of the Coronavirus Disease (COVID-19). As one’s language can provide insight in one’s mental health, this pre-registered study analyzed word use in a DV online support group, aiming to investigate the impact of the COVID-19 pandemic on DV victims in an ex post facto research design. Words reflecting social support and leisure activities were investigated as protective factors against linguistic indicators of depression in 5,856 posts from the r/domesticviolence subreddit and two neutral comparison subreddits (r/changemyview & r/femalefashionadvice). In the DV support group, the average number of daily posts increased significantly by 22% from pre- to mid-pandemic. Confirmatory analysis was conducted following a registered pre-analysis plan. DV victims used significantly more linguistic indicators of depression than individuals in the comparison groups. This did not change with the onset of COVID-19. The use of negative emotion words was negatively related to the use of social support words (Spearman’s rho correlation coefficient [rho] = −0.110) and words referring to leisure activities (rho = −0.137). Pre-occupation with COVID-19 was associated with the use of negative emotion words (rho = 0.148). We conclude that language of DV victims is characterized by indicators of depression and this characteristic is stable over time. Concerns with COVID-19 could contribute to negative emotions, whereas social support and leisure activities could function to some degree as protective factors. A potential weakness of this study is its cross-sectional design and the lack of experimental control. Future studies could make use of natural language processing and other advanced methods of linguistic analysis to learn about the mental health of DV victims.

While the 2019 Coronavirus Disease (COVID-19) pandemic has a severe impact on people’s physical health, causing more than 6.5 million deaths as of September 2022, lockdowns and quarantine also negatively impacted people’s mental health (Brooks et al., 2020; Hopkins, 2020; Paredes et al., 2021). Victims of domestic violence (DV) have been particularly vulnerable for developing mental health problems, as government-mandated lockdowns forced them to spend more time with their abusers and reduced their possibilities to escape or to receive help (Da Silva et al., 2020).

## Domestic Violence and COVID-19

DV can be defined as physical, sexual, or emotional abuse between intimate partners, family members, or people living in the same household (Karystianis et al., 2020). The lifetime prevalence of experiencing physical or sexual partner violence differs per country and is reported to be between 15% and 71% (Garcia-Moreno et al., 2007). Nevertheless, it is considered one of the most underreported crimes Europe wide with only 14.5% of incidents being reported to the police and a much greater number of unknown cases (FRA, 2014). With DV severity increasing, physical and mental health functioning declines (Straus et al., 2009). A review of 66 studies revealed that suffering from DV is associated with depression, post-traumatic stress disorder, anxiety, sleep disorders, and suicidal thoughts (Dillon et al., 2013). According to the FRA (2014), 27.5% of women state to experience depression and 38.5% indicate anxiety symptoms as a consequence of intimate partner violence.

The pandemic further intensified DV victims threatening home situations with 52% reporting a negative influence on their mental health and abuse coping mechanisms (Office for National Statistics, 2020). COVID-19 increased financial insecurity, scarcity of basic resources and routine disruptions which have all shown to be interdependent causes for intimate partner violence (Jewkes, 2002). Moreover, not only did long- term social distancing increase threat exposure to predators and limit social support resources (Office for National Statistic, 2020), but it also increased other risk factors such as domestic alcohol and drug consumption (Da Silva et al., 2020). Power theory of domestic violence proposes that predators execute violence to gain power and control through physical, emotional and sexual abuse. Modeled in early family structures, many predators see violence as an acceptable tool to express conflict and release frustration (Burelomova et al., 2018). Given that not only victims but also predators were affected by disruptions of daily rhythms and uncertainty about the future, one can expect that more violence was exerted with the aim of regaining control for something in their life.

Various nationwide surveys show an increase of 30% to 46% in DV since the onset of COVID-19 in England (Office for National Statistic, 2020), India (Pattojoshi et al., 2021), France, Spain, and Poland (United Nations [UN] Development Programme, 2020). Even more, where DV already occurred in India, its severity increased by 77.6%, with 22.8% of victims reporting difficulties to reach out for help due to COVID-19 (Pattojoshi et al., 2021). Increased barriers to reach out for help in victims who already show one of the lowest reporting rates could reflect victims helplessness and limited resources. COVID-19 measures significantly limited the availability to engage in coping mechanisms such as social support, therapeutic counseling, exercising or escaping the predator through engagement in leisure activities. Allostatic load describes the inability to efficiently cope and adapt to cumulative stressors and is considered a major risk factor for developing symptoms of major depressive disorder (McEwen, 2003). COVID-19 has caused significant lifestyle changes that impose excessive challenges not only for people in the general population, but especially for DV victims. Increasing exposure to pre-existing stressors of violence, reducing protective factors and experiencing additional stressors such as worry about financial stability may exceed one’s ability to cope.

Social support has long been argued to be a strong protective factor against the negative influences of stressors on mental health outcomes. The stress buffering hypothesis states that social support increases people’s self-esteem and stimulates reappraisal of stressful events (Cohen & McKay, 2020). Likewise, the evolutionary theory of loneliness emphasizes that isolation increases negative appraisal of emotions and threat (Cacioppo & Cacioppo, 2018). A recent study has in fact shown that high loneliness and low social support were both predictors of adverse mental health outcomes during the COVID-19 pandemic (Rossi et al., 2020).

## Language Use and Mental Health

While retrospective surveys on the effects of the pandemic on mental health provide important insights, they are limited by human biases in memory and perception (Hardt & Rutter, 2004; Wilson et al., 2003). Language was shown to be a powerful tool to illustrate writers’ emotion processing and affective mental states (Tausczik & Pennebaker, 2010). Analyzing posts on social media platforms such as Reddit provides the possibility to get a less biased view into the past while also allowing for investigating emotions without threat to reactivity of assessment. On Reddit, people can discuss topics of personal concern (Low et al., 2020; Ronner & Linkowski, 2020), such as political opinions, fashion, or their experiences with DV. Word use has the potential to disclose the author’s underlying cognitive mechanisms such as attentional focus, reasoning, emotional processing, and intentions (Tausczik & Pennebaker, 2010). These cognitive mechanisms in turn reflect mental well-being (Tausczik & Pennebaker, 2010). Analyzing historical testimonies should be less subject to the earlier mentioned memory and perception biases and simultaneously creates the opportunity to gather data on a large sample. For an objective evaluation, the linguistic features of these testimonies can be extracted.

Cognitive models propose that depressive symptoms are marked by negative appraisal, attention bias, and self-referential rumination (Beck, 2002; Pyszczynski & Greenberg, 1987). The emotion words and pronouns we use in daily life communication such as diary entries reflect these cognitive mechanisms and give insight into writers’ mental states. Cohn et al. (2004) discovered an increase in negative emotions, psychological distance, social processes, and cognitive processes in diary entries immediately after the September 11 attacks. Word use has also been associated with psychopathology. Psychiatric patients referred in essays about their life’s more to the self and to negative emotions, but less to positive emotions than healthy individuals. This reflects a high degree of self-focus and negative cognitions common for many psychological disorders (Molendijk et al., 2010; Pyszczynski & Greenberg, 1987). Further, the use of first-person singular and negative emotion words has repeatedly been associated with depression (Rude et al., 2004; Tadesse et al., 2019). This can be explained with cognitive theories of depression postulating that depression-prone individuals can be characterized by negative thinking styles and a tendency to focus on the self, especially when confronted with stressors (Beck, 2002; Pyszczynski & Greenberg, 1987). These maladaptive cognitions affect mood especially when triggered by stress (Beck, 2002).

Similarly, linguistic markers have shown to predict treatment outcomes. Patients with personality disorders undergoing treatment show a decreased use of first-person singular, negative emotions, causation words, past, and future tense, while showing an increase in positive emotions and present-tense words. A reduced use of negative emotion and negation words in turn predicts a favorable treatment outcome (Arntz et al., 2012). Correspondingly, cognitive processing words such as insight and causation words extracted from online anorexia nervosa forums have been associated with a lack of rationalization about the disorder’s negative health consequences, avoidance behavior and maladaptive coping (Lyons et al., 2006).

Recent statistical analysis of COVID-19 consequences has shown a sharp increase in both depression symptoms and DV reports worldwide (Rossi et al., 2020; Salari et al., 2020). As many DV cases go undetected, it is especially difficult to investigate their mental wellbeing and emotional states unless victims come forward. Nevertheless, reports have shown a tremendous increase of people seeking DV information online. The UK National Domestic Abuse Helpline reports a 700% increase of website visits between April and June 2020 (Office for National Statistics, 2020). Writing anonymous posts about experiences in online support forums may present a lower threshold for DV victims compared to seeking help at healthcare facilities. Making use of these insights, the current study investigates how COVID-19 affects the mental well-being of DV victims by analyzing the linguistic properties of their testimonies written before and during the COVID-19 pandemic in an ex post facto design. This is done by investigating the following hypotheses:

(1) Posts written by victims of DV show a higher number of linguistic markers of depression, especially during COVID-19, than the general population.
(2) DV victims who use more words indicative of social support or leisure activities display fewer linguistic indicators of depression.
(3) The use of words related to COVID-19 is positively associated with the use of linguistic markers of depression.

## Methods

The study received approval from the research ethics committee of the psychological institute of Leiden University (number: 2021-03-30-M.L.-Molendijk-V2-3146).

### Data Collection

Data was collected from the subreddits r/domesticviolence, r/changemyview, and r/femalefashionadvice. R/domesticviolence offers “information and support for victims, survivors, their family and friends.” R/changemyview was chosen as a neutral comparison group because its members discuss personal opinions about political and social issues. As the majority of DV victims are female (Straus et al., 2009), it could be expected that r/changemyview has more male authors than r/domesticviolence. It is not possible to extract the author’s gender of most posts. To control for the potential confounding variable of gender, we selected r/femalefashionadvice as another comparison group as it is expected to have mostly female authors. Comparison groups were similar in posting frequency and post length to r/domesticviolence. Demographical analyses have shown that 69% of Reddit users are male and 56% are between 18 and 29 years of age (Barthel et al., 2016), with 50% of Reddit traffic coming from the US, 8% from the UK, and 8% from Canada (Tankovska, 2021).

Posts from all subreddits were collected from January to June 2020 to capture the onset of the COVID-19 pandemic. Posts were also collected from January to June 2019, which served as a control period. During these periods of observation, 2,454 posts have been written in r/domesticviolence. The r/changemyview and the r/femalefashionadvice subreddit have more members and consequently contained more posts. To ensure a balanced sample 2,454 posts were randomly drawn from these subreddits by using the random sampling function of the pandas python library (The Pandas Development Team, 2021). For all three subreddits, posts were separated into the groups “pre-pandemic” (posts written up to the 10th of March 2020) and “post-pandemic” (posts written from the 20th of March 2020 onward) to compare word use among both timeframes. Posts written from 11th to 19th of March 2020 were excluded from analysis because of variations in lockdown onset across different regions. To compare pre- and mid-pandemic posts to a baseline level, the posts written in 2019 were separated in a similar way.

The Pushshift Reddit API (Baumgartner & Seiler, 2019) was used to automatically retrieve posts from Reddit. In a human annotation task, the posts from the r/domesticviolence subreddit were coded on: (I) whether a post was written by the victim or by a third person, (II) whether the victim is still in a relationship with the abuser or not, and (III) the gender of the author. Duplicates were removed. After randomly selecting only one post per user to ensure independent observations, 5,856 posts (1,869 from r/domesticviolence, 2,134 from r/changemyview, 1,853 from r/femalefashionadvice) were included in the final dataset.

### Linguistic Analysis

All posts collected were analyzed for linguistic features using the Linguistic Inquiry and Word Count 2015 (LIWC2015) software. The LIWC is a language analysis program that counts words of pre-defined linguistic categories and returns for each category a percentage of how often the category is represented in each text (Pennebaker et al., 2015). It consists of over 90 variables capturing linguistic dimensions such as pronouns, affective processes, and summary variables, built from a dictionary of over 6,000 words. The LIWC has been extensively validated and was shown to appropriately reflect the author’s underlying cognitive processes (Tausczik & Pennebaker, 2010). Even though it fails to consider the context in which words are used, the LIWC scores of texts written in an online breast cancer support group showed moderate correlations with the scores given by human judges (Alpers et al., 2005). The LIWC variables have reasonable internal consistency (Pennebaker et al., 2015).

The percentage of first-person singular words in a text was used to represent a focus on the self, and the percentage of negative emotion words to represent negative sentiment. Further, the LIWC variable indicating leisure activities was analyzed, and in addition to the standard LIWC categories, two categories were manually added. The first reflected how much a text refers to terms associated with the COVID-19 pandemic and contains words such as “Covid,” “lockdown,” and “pandemic.” The second manually created variable reflects the percentage of words referring to social support and contains words such as “understands,” “helps” and “trust.”

For exploratory analysis, the LIWC variables representing a focus on the past, a focus on the future, cognitive insight, affiliation, negation words, and analytical thinking were used. Analytical thinking is a summary variable calculated by the LIWC and defined by displaying formal, logical, and hierarchical thinking patterns (Pennebaker et al., 2015). An overview of the linguistic features analyzed in this study can be found in the online supplement. Further, an emotional distancing variable consistent of low self-occupation words, increased third person singular pronouns, past tense use, long words, and increased use of articles was calculated (Cohn et al., 2004; Tausczik & Pennebaker, 2010).

Posts that contained less than 50 words or had less than 50% of their words recognized by the LIWC were excluded, since these posts may not provide reliable estimates (Pennebaker et al., 2015).

### Data Analysis

All data analyses were performed in R version 4.0.2. Confirmatory analysis was conducted as specified in the pre-analysis plan. All code used for data collection, pre-processing and analyses was uploaded on the study’s OSF project page (https://osf.io/ckyus).

An ANOVA was run predicting daily posting frequency with subreddit and timeframe as fixed factors. All assumptions were either met or the model was robust to a violation of the assumption due to large and homogenous group sizes. For post-hoc comparisons, Student’s t-test was used to compare pre- to mid-pandemic posts and Cohen’s d was used as a measure of effect size.

A MANCOVA was run with subreddit and time as fixed-factors and the use of negative emotion and first-person singular words as dependent variables. The percentage of words recognized by the LIWC was included as a covariate to account for potential demographical differences between the subreddits. The assumption of absence of multicollinearity was met, while the assumptions of a multivariate normal distribution of the dependent variables, homogenous variances, and a linear relationship between the covariate and the dependent variables with parallel slopes for all groups of the independent variables were not met. To ensure robustness to the violation of homogeneity of variances, all groups were randomly subsampled to 1.5 times the size of the smallest group, after which 4,776 observations were available for analysis. Non-linear transformations were applied to better satisfy the assumption of a multivariate normal distribution. The violations of the other assumptions should not interfere with statistical analysis since the large sample size of at least 262 observations per group generates robustness to these violations (Hand & Taylor, 1987). The assumption of independent observations is expected to be met as only one post per user was analyzed. Partial eta-squared was used as a measure of effect size of the main and interaction effects. The a-priori comparisons were specified in the pre-registration and tested with Wilcoxon Rank-Sum tests. The Hodges-Lehmann estimator was used as a measure of effect size and Bonferroni correction was applied for multiple testing.

Spearman’s rho was used to test for associations between social support and leisure activities with linguistic indicators of depression. Only posts from the subreddit r/domesticviolence written by the victims themselves were used. The hypothesis that an increased concern with COVID-19 goes along with more linguistic indicators of depression was tested using non-parametric correlation analyses. Expecting positive associations, posts from all subreddits and all users were used. Bonferroni correction was applied to control for multiple testing. The three subreddits were compared on the emotional distancing variable using Wilcoxon Rank-Sum tests. Analyses were repeated without excluding posts of less than 50 words or where less than 50% of words were recognized by the LIWC.

## Results

After applying the inclusion criteria, 5,288 posts (1,779 from r/domesticviolence, 2,079 from r/changemyview, 1,430 from r/femalefashionadvice) were available for statistical analysis. From the 1779 posts in r/domesticviolence, 1,267 posts were written by a victim and 378 posts by another person familiar to the victim. For 134 posts the position of the author could not be identified. In 881 cases, the victim was in the abusive relationship when writing the post, and in 637 cases the victim had left the abusive relationship. In 261 cases the authors persistence in the relationship was unknown. Three hundred sixty-nine posts were written by a female author, 104 by a male author, and from 1,306 posts the gender of the author could not be determined. Descriptive statistics on the length of the posts, the words captured by the LIWC, and outcome variables are provided in Table 1.

**Table 1.** Mean (*SD*) of LIWC Variables by Subreddit.

Word count		% Recognized	% Neg. emotions	% First person sing.
r/domesticviolence	395 (431)	92.76 (5.17)	4.27 (2.19)	9.13 (3.89)
Written by victim	429 (455)	93.46 (3.00)	4.35 (2.02)	10.62 (2.87)
… by third party	389 (388)	93.33 (3.59)	3.94 (1.77)	6.60 (3.35)
r/changemyview	305 (284)	84.86 (6.86)	2.57 (1.99)	2.88 (2.58)
r/femalefashionadvice	122 (132)	79.57 (11.52)	1.01 (1.37)	6.20 (3.57)

An ANOVA predicting daily posting frequency from subreddit and time revealed an interaction effect (*F*(6, 1021) = 19.08, *p* < .001, *η*^2^ = 0.10). Post-hoc comparisons showed that the number of posts per day increased by 22% in r/domesticviolence during the pandemic, which was significantly more than pre-pandemic (*t*(154) = 4.01, *p* < .001, 95% CI [0.94, 2.78]; Supplemental Figure 1). Daily posting frequency in r/changemyview increased by 46% (*t*(127) = 10.56, *p* < .001, 95% CI [32.69, 47.77]), while the number of daily posts in r/femalefashionadvice decreased by 8% during the pandemic (*t*(185) = −3.27, *p* = .001, 95% CI [−4.95, −1.23]).

### Confirmatory Analysis

We observed significant main effects of time (*F*(6, 8040) = 16.58, *p* < .001, *η*^2^ = 0.002) and of subreddit (*F*(4, 8040) = 1704.80, *p* < .001, *η*^2^ = 0.35) on the use of first person singular and negative emotion words. The interaction effect between time and subreddit was significant (*F*(12, 8040) = 2.19, *p* = .01, *η*^2^ = 0.003) on the use of first-person words (*F*(6, 4021) = 2.91, *p* = .01) but not for negative emotions (*F*(6, 4021) = 1.50, *p* = .17). Means and standard deviations of the dependent variables per subreddit and timeframe are displayed in Table 2.

**Table 2.** Mean (*SD*) of Negative Emotions (NE) and First Person Singular (first PS) Use Per Subreddit Over Time.

		Pre-pandemic			Mid-pandemic
		Winter 2019	Spring 2019	Winter 2020	Spring 2020
r/domesticviolence	NE	4.507 (1.990)	4.282 (1.940)	4.076 (1.896)	4.305 (1.862)
	First PS	10.450 (2.785)	10.355 (2.645)	10.884 (2.774)	10.578 (2.815)
r/changemyview	NE	2.453 (1.727)	2.468 (1.849)	2.429 (1.844)	2.718 (2.018)
	First PS	6.992 (3.004)	6.743 (3.122)	6.907 (3.240)	6.359 (3.531)
r/femalefashionadvice	NE	1.042 (1.098)	1.153 (1.252)	0.998 (1.148)	1.114 (1.327)
	First PS	2.744 (2.396)	2.757 (2.510)	2.911 (2.601)	2.910 (2.503)

Significantly more negative emotions and first-person singular words were used in mid-pandemic posts in r/domesticviolence than in r/changemyview (negative emotions: *W* = 116,414, *p* < .001, *d* = 1.60, 95% CI [1.45, Inf]; first-person singular: *W* = 149,307, *p* < .001, *d* = 7.83, 95% CI [7.54, Inf]) and in r/femalefashionadvice (negative emotions: *W* = 113,693, *p* < .001, *d* = 3.21, 95% CI [3.02, Inf]; first-person singular: *W* = 101,730, *p* < .001, *d* = 4.36, 95% CI [3.94, Inf]). The use of negative emotions and first-person singular in r/domesticviolence was not significantly higher pre versus mid-pandemic (negative emotions: *W* = 156076, *p* = .72, *d* = 0.12, 95% CI [−0.05, Inf]; first-person singular: *W* = 149,432, *p* = 1, *d* = −0.01, 95% CI [−0.20, Inf]).

Posts with fewer negative emotion words contained more words referring to social support and leisure activities. No significant correlations were found between the use of first-person singular with words indicative of social support or leisure activities (Table 3). A higher concern with COVID-19 was associated with using more negative emotion words, but not with using first-person singular (Table 3). Results were not different if case analyses were run with posts of less than 50 words included or when the analyses were not controlled for the percentage of words that were captured by the LIWC dictionary.

**Table 3.** Correlations of Linguistic Indicators of Depression with Possible Protective Factors and COVID-19.

		Social support^a^	Leisure^a^	Concern with COVID-19^b^
Negative emotions	rho	−.110	−.137	0.148
	95% CI	[−0.169, −0.050]	[−0.197, −0.078]	[0.123, 0.173]
	*p*-value	<.001*	<.001*	<.001*
First-person singular	rho	0.033	−.050	0.011
	95% CI	[−0.024, 0.094]	[−0.111, 0.011]	[−0.016, 0.040]
	*p*-value	1	.179	.404

*Note*. Bonferroni correction was applied for multiple testing; *significant at alpha <.01; Rho refers to Spearman’s rho correlation coefficient.

a Using only posts written by victims of DV.

b Using all posts from all subreddits.

### Exploratory Analysis

Some non-hypothesized, but noteworthy patterns were observed in the data. The use of negative emotions and first-person singular were negatively correlated with analytical thinking (rho = −0.33, *p* < .001, 95% CI [−0.35, −0.31]; rho = −0.61, *p* < .001, 95% CI [−0.62, −0.59]) and positively with a focus on the past (rho = 0.33, *p* < .001, 95% CI [0.31, 0.35]; rho = 0.37, *p* < .001, 95% CI [0.35, 0.39]) and the future (rho = 0.17, *p* < .001, 95% CI [0.15, 0.19]; rho = 0.14, *p* < .001, 95% CI [0.12, 0.17]). In turn, analytical thinking was negatively correlated with focusing on the past (rho = −0.36, *p* < .001, 95% CI [−0.37, −0.33]) and the future (rho = −0.18, *p* < .001, 95% CI [−0.20, −0.15]; Supplemental Table 4).

Posts in the r/domesticviolence subreddit contained 17% more emotional distancing words than posts in the r/femalefashionadvice subreddit (Median r/domesticviolence = 34.15, Median r/femalefashionadvice = 29.31, *W* = 870,737, *p* < .001), and 5% less than posts in the r/changemyview subreddit (Median r/changemyview = 35.78, W = 165,5041, *p* < .001).

Individuals who left the abusive relationship used more insight words (*W* = 319,980, *p* < .001, *d* = 0.30, 95% CI [0.18, 0.43]) and displayed more analytical thinking (*W* = 339,744, *p* < .001, *d* = 5.93, 95% CI [4.31, 7.57]). Individuals who stayed in the abusive relationship used more negations (*W* = 239,712, *p* < .001, *d* = −0.42, 95% CI [−0.59, −0.25]) and words indicative of affiliation (*W* = 232,571, *p* < .001, *d* = −0.32, 95% CI [−0.43, −0.20]; Supplemental Table 5).

## Discussion

This study found that victims of DV display more linguistic indicators of depression than individuals in two comparison groups. While these indicators did not increase with the COVID-19 pandemic, more posts were submitted in the DV online support group and r/changemyview during the pandemic, but not in r/femalefashionadvice. Displayed negative emotions were positively associated with preoccupation with COVID-19 and negatively with references to leisure and social support. Such associations could not be found for first-person singular word use. Linguistic indicators of depression were positively associated with a focus on the past and the future, but negatively with analytical thinking. DV victims who left their abusive relationship used more words indicative for analytical thinking and cognitive insight and fewer negations and affiliation words than victims who stayed in the abusive relationship.

The relatively high use of linguistic indicators of depression in the DV support group suggests a victim-typical language characterized by negative emotion words and a high focus on the self. This goes in line with research suggesting an association between suffering from DV and mental health issues such as depression (Dillon et al., 2013). These characteristics were independent of the COVID-19 pandemic and hence seem stable over time.

The increase in number of posts in the DV online support group and r/changemyview during the Covid-19 pandemic could be explained by a general increase in social media use, as was previously the case during natural disasters (Niles et al., 2019). Since posting frequency did not increase in r/femalefashionadvice, it could also suggest that cases and severity of DV became even more problematic than before, as reported by UN Women (2020) and Pattojoshi et al. (2021). This is supported by data from the UK National DV Helpline Website Refuge which reported a 700% increase of website visits (Office for National Statistics, 2020). Posting frequency in r/changemyview might have increased due to the relevance of other issues discussed in this subreddit. Future research should investigate whether and to which extend social media use increased during the pandemic.

The use of social support and leisure words was negatively correlated with the use of negative emotion words. This provides evidence for the hypothesis that social support and leisure activities could shield victims to some degree from the negative psychological consequences of DV which is in line with the Stress Buffering Hypothesis, the Evolutionary Theory of Loneliness (Cacioppo & Cacioppo, 2018), and with research which found that social support (Eagle et al., 2019) and leisure activities (Lu, 2011) are negatively associated with depression. Moreover, this finding may indicate that those without social support and more negative emotion word use are more likely to reach out for help in online forums. As has also been hypothesized, the more references to COVID-19 were made, the more often negative emotion words were used. This demonstrates a possible impact of being concerned with the COVID-19 pandemic on one’s mental health, as has also been suggested by Ding et al. (2021), Röhr et al. (2020), and Salari et al. (2020). The hypotheses that self-focus would be negatively associated with social support and leisure, but positively associated with words referring to COVID-19 could not be confirmed. Possibly, one’s level of self-focus might not directly translate into more use of first-person singular words. Another potential interpretation would be that although less social support, less leisure, and more concern with COVID-19 might contribute to more negative emotions, they might not directly activate a maladaptive cognitive pattern including a focus on the self.

Moreover, linguistic analysis of diary entries from 9/11 survivors showed an increased use of words reflecting psychological distancing (words longer than six letters; decreased first-person singular pronouns; decreased present-tense use; increased use of articles) (Cohn et al., 2004). Post-traumatic stress disorder is among the most frequently occurring psychopathologies among DV victims with detachment and numbing being core diagnostic symptoms (American Psychological Association, DSM-5 Task Force, 2013). Therefore, the absence of an increase in self-focus may be explained by cognitive alterations caused by trauma of r/domesticviolence users. This is supported by our findings that the word use of domestic violence victims reflects higher levels of emotional distancing than that of non-victimized women from r/femalefashionadvice.

Linguistic indicators of depression were associated with a focus on the past, on the future, and with less analytical thinking. This aligns well with cognitive theories of depression (Beck, 2002; Pyszczynski & Greenberg, 1987). These theories emphasize that depressed individuals often ruminate over the past and have negative expectations about the future. Nevertheless, in the context of domestic violence, victims might also avoid thoughts about the present characterized by abuse by thinking about good times in the past or prospects of a better future. The negative correlations found between the linguistic indicators of depression and analytic thinking suggest that maladaptive cognitions experienced by depressive individuals do not follow a rationale.

Victims who left their abusive relationship showed different linguistic patterns than victims who stayed. The higher levels of analytical thinking displayed by victims who left their abusive relationship could be interpreted in two manners: Either that leaving an abusive relationship frees individuals from constraints and allows them to engage in more introspective thinking, or that thinking logically and hierarchically might motivate someone to leave an abusive situation. While likely a combination of both interpretations applies, the second interpretation can be underlined by research indicating that maladaptive cognitions such as denial, self-blame, or guilt disable victims from reaching out for help and reduce readiness for change (Buel, 1999; Heise et al., 1999).

The increased use of cognitive insight words by the same individuals is in line with the findings of Homan et al. (2020). They found that victims who left an abusive relationship often used phrases such as “realize deserve better,” suggesting that increased awareness of fairness and justice are relevant factors in leaving abusive relationships (Homan et al., 2020). The higher use of affiliation words by victims who stayed in their abusive relationship points out that being more affiliated to someone (“I feel like I need him and I want him”) likely contributes to staying in the relationship. Meanwhile, the high use of negations by the same individuals might indicate that missing financial or social support (“I don’t have any independent financial income,” “I’ve got no friends that would let me stay with them,” “I don’t know who I can go to”) or fearing certain outcomes (“I don’t want my baby seeing this,” “I don’t want my friends or family to know”) can keep someone from escaping DV. Therefore, having financial stability and an awareness of fairness and justice may help DV victims in leaving abusive relationships. Future research should investigate these exploratory findings in a confirmatory manner.

Our results emphasize the urgent need for DV prevention and low threshold support contact points during government-mandated stay-at-home orders and demonstrate the impact of personal COVID-19 concern on one’s mental health. Moreover, the results suggest social support and leisure activities as protective factors against the negative psychological consequences of DV victimization.

### Limitations

Limitations of the current study include the following. As the analyzed testimonies were collected from an online support group, they were mostly written by individuals who recognized their victimization and engaged in problem-focused coping by actively reaching out for social support. In this sense, members in r/domesticviolence can be assumed to be posting with an inherently different motivation than members of both comparison groups, which could have confounded the reported results. Research by Pennebaker (2004) shows that expressive writing about emotional events may function as a therapeutic tool by confronting and structuring maladaptive thoughts. Therefore, the experimental group’s mental health condition may thereupon be improved due to writing itself. Overall, it can be assumed that r/domesticviolence does not encompass all types of DV victims. Many victims might not recognize their victimization, might be too young, too ashamed, or unable to reach out for help (Cho et al., 2020; Overstreet & Quinn, 2016). These victims might especially suffer from additional burdens such as DV stigmatization. Consequently, the current study might not capture the full relationship between DV, COVID-19, and mental health because it investigated a selective sample. Likewise, as not all DV victims have access to the internet and social media, this reduces the generalizability of this study. Such an online data collection method, however, has the benefit of targeting a group of people that would have remained understudied in a non-anonymous offline setting as they might not be able to express themselves otherwise.

Further, analyzing anonymous testimonies shared on social media limited the ability to investigate and correct for sociodemographic differences between the comparison groups, as no sociodemographic data was available. Additionally, for many posts it could not be investigated what type or severity of DV was experienced, whether posts are indeed written by first-hand experiencers, and how long after the experience they were written. As not all individuals posting in English may be native English speakers, analyzing their posts might not be as informative as assumed.

Our results may be limited by the validity of the LIWC. While the LIWC offers the possibility to objectively analyze written texts, it counts words without considering the context they appear in. Since the meaning of words is often context-dependent, the LIWC can fail to extract the true meaning of a text and might focus too much on the specified target expressions. Nevertheless, moderate correlations have been observed between LIWC ratings and the evaluations of human judges (Alpers et al., 2005). Since reports about the occurrence of DV by the nature of their content carry a rather negative sentiment, this sentiment might not directly translate to the victim’s general perception of life. To counteract these limitations, future research is encouraged to utilize more advanced methods of linguistic analysis such as the Meaning Extraction Method (Chung & Pennebaker, 2008) or Stanford’s Natural Language Toolkit (Bird et al., 2009), to investigate a more diverse set of language sources, and to investigate individuals’ change in language use over time.

## Conclusion

The current study has demonstrated that linguistic analysis of narratives from online support groups can provide insights into a group’s mental well-being, needs, and influential factors. Due to the LIWC’s context-independent scoring system, future research should include more advanced methods of linguistic analysis. To capture a more diverse set of observations, further research is advised to not only rely on online support groups, but to also analyze essays, natural dialogs, or interviews. The insights generated in this study could be used to improve crisis-response management. When taking measures to restrict the spread of a disease, the protection of vulnerable groups such as DV victims should be ensured. While minimizing possible anxieties about diseases such as COVID-19, possibilities to seek out social support and to engage in leisure activities should be maximized.

## Supplemental Material

sj-docx-1-sgo-10.1177_21582440221146135 – Supplemental material for Linguistic Analysis of Online Domestic Violence Testimonies in the Context of COVID-19 Click here for additional data file. Supplemental material, sj-docx-1-sgo-10.1177_21582440221146135 for Linguistic Analysis of Online Domestic Violence Testimonies in the Context of COVID-19 by Valentin L. Buchner, Sharina Hamm, Barbara Medenica and Marc L. Molendijk in SAGE Open
