# Supplementary material for: Linguistic Analysis of Online Domestic Violence Testimonies in the Context of COVID-19
Source: Sage Open. 2023 Jan 10;13(1):21582440221146135. doi: 10.1177/21582440221146135 (PMC9834616; doi:10.1177/21582440221146135)
Supplement: sj-docx-1-sgo-10.1177_21582440221146135 – Supplemental material for Linguistic Analysis of Online Domestic Violence Testimonies in the Context of COVID-19 [file sj-docx-1-sgo-10.1177_21582440221146135.docx]

# Appendices. Online supplement

## Appendix A*.* Linguistic Properties and Definitions

**Table 4**

*Meaning of the linguistic properties analyzed in this study*

| Variable | Example words | Meaning |
| --- | --- | --- |
| WC |  | Word Count, the number of words contained in the text |
| Dic |  | Percentage of words recognized by the LIWC dictionary |
| Analytic |  | Summary variable indicating the degree of formal, logical, and hierarchical thinking patterns |
| Emotional distancing |  | Summary variable representing low use of self-occupation words, increased use of third person singular pronouns, past tense, long words, and articles |
| i | I, me, mine | Percentage of words which are first-person singular pronouns |
| negemo | hurt, ugly, nasty | Percentage of words which are referring to negative emotions |
| negate | no, not, never | Percentage of words which are negations |
| insight | think, know | Percentage of words which indicate cognitive insight |
| focuspast | ago, did, talked | Percentage of words indicating a focus on the past |
| focusfuture | may, will soon | Percentage of words indicating a focus on the future |
| leisure | cook, chat, movie | Percentage of words which are referring to leisure activities |
| affiliation | ally, friend, social | Percentage of words indicating affiliation |
| socsupport | understands, helps, trust | Percentage of words that appear in the manually created “socsupport” dictionary |
| Covidconcern | Covid, lockdown, pandemic | Percentage of words that appear in the manually created “Covidconcern” dictionary |

Terms contained in “Covidstrict”:

Corona, Coronavirus, Covid, COVID-19, COVID19, face-mask, mask, lockdown, Quarantine, Virus, Curfew, Pandemic, Self-quarantine, Self-Isolation, Super-spreader, Vaccine , Contagious, Contact, Ill, Sick, Fever, Cough, Tiredness, Tired, Disinfected, Infected, Infection, Closed, Hygiene, Hand-washing, Hand-gel, Sanitiser, SARS-CoV-2, Respiratory, Test, Testing, Tested, Cases, Numbers, Rules, Measures, ICU, Regulations, Virologist, Mutation, Immunity, Vaccination, Prevention, Sars, Influenca, Distancing, Ventilator, Symptomatic, Asymptomatic, Epidemiolog*, Immun*, Transmission

Terms contained in “socsupport”:

Support*, Friend*, Encourag*, Help*, Warm*, Guid*, Trust*, Comfort*, Together, Assist*, Rely, Relies, Listen*, Understand*, Shar*, Talk*

## Appendix B. Supplement to Exploratory Analysis

**Table 4**

*Correlations among linguistic indicators of depression, past- and future focus, and analytical thinking*

|  |  | Analytical thinking | First-p. sing. | Negative emotions | Focus on past | Focus on future |
| --- | --- | --- | --- | --- | --- | --- |
| AT | rho | 1.000 | -.606 | -.331 | -.355 | -.175 |
|  | 95% CI |  | [-.621, -.590] | [-.353, -.309] | [-.374, -.333] | [-.200, -.153] |
| FPS | rho |  | 1.000 | .249 | .373 | .143 |
|  | 95% CI |  |  | [.226, .271] | [.351, .394] | [.121, .166] |
| NE | rho |  |  | 1.000 | .329 | .170 |
|  | 95% CI |  |  |  | [.306, .352] | [.145, .194] |
| FP | rho |  |  |  | 1.000 | .107 |
|  | 95% CI |  |  |  |  | [.083, .132] |

*Rho refers to Spearman’s rho correlation coefficient*

*All correlations are significant at alpha < .001*

**Table 5**

*Characteristics of posts in r/domesticviolence depending on victim’s persistence in relationship*

|  |  | Mean (SD) | | |
| --- | --- | --- | --- | --- |
|  |  | stayed |  | left |
| Cognitive insight |  | 2.253 (1.203) |  | 2.552 (1.397) |
| Analytical thinking |  | 23.680 (15.862) |  | 29.689 (18.074) |
| Affiliation |  | 3.229 (1.933) |  | 2.728 (1.678) |
| Negations |  | 2.525 (1.267) |  | 2.183 (1.165) |

| *All differences have p-values < .001 tested with Wilcoxon rank sum test* |
| --- |

**Figure 1**

*Posting frequency over time per subreddit*


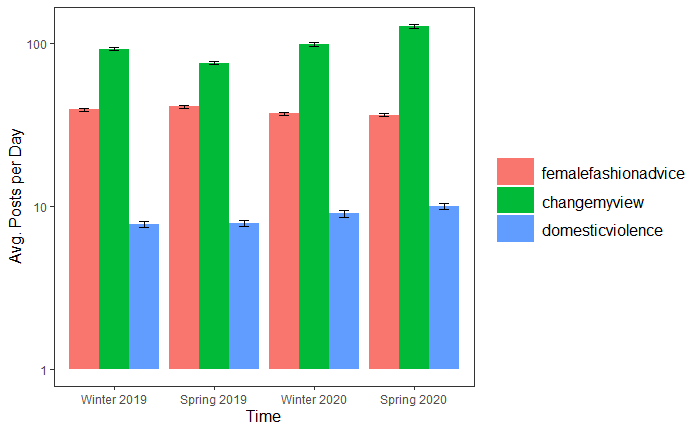


|  |
| --- |

| Word count | | % Recognized | % Neg. emotions | % 1^st^ person sing. | |
| --- | --- | --- | --- | --- | --- |
| r/domesticviolence | 395 (431) | 92.76 (5.17) | 4.27 (2.19) | | 9.13 (3.89) |
| Written by victim | 429 (455) | 93.46 (3.00) | 4.35 (2.02) | | 10.62 (2.87) |
| … by third party | 389 (388) | 93.33 (3.59) | 3.94 (1.77) | | 6.60 (3.35) |
| r/changemyview | 305 (284) | 84.86 (6.86) | 2.57 (1.99) | | 2.88 (2.58) |
| r/femalefashionadvice | 122 (132) | 79.57 (11.52) | 1.01 (1.37) | | 6.20 (3.57) |

|  | |  |  | Pre-pandemic | | |  | Mid-pandemic |
| --- | --- | --- | --- | --- | --- | --- | --- | --- |
|  |  | |  | Winter 2019 | Spring 2019 | Winter 2020 |  | Spring 2020 |
| r/domesticviolence | | NE |  | 4.507 (1.990) | 4.282 (1.940) | 4.076 (1.896) |  | 4.305 (1.862) |
|  | | 1^st^ PS |  | 10.450 (2.785) | 10.355 (2.645) | 10.884 (2.774) |  | 10.578 (2.815) |
| r/changemyview | | NE |  | 2.453 (1.727) | 2.468 (1.849) | 2.429 (1.844) |  | 2.718 (2.018) |
|  | | 1^st^ PS |  | 6.992 (3.004) | 6.743 (3.122) | 6.907 (3.240) |  | 6.359 (3.531) |
| r/femalefashionadvice | | NE |  | 1.042 (1.098) | 1.153 (1.252) | 0.998 (1.148) |  | 1.114 (1.327) |
|  | | 1^st^ PS |  | 2.744 (2.396) | 2.757 (2.510) | 2.911 (2.601) |  | 2.910 (2.503) |

|  |  |  | Social support^a^ |  | Leisure^a^ |  | Concern with COVID-19^b^ |
| --- | --- | --- | --- | --- | --- | --- | --- |
| Negative emotions | rho |  | -.110 |  | -.137 |  | .148 |
|  | 95% CI |  | [-.169, -.050] |  | [-.197, -.078] |  | [.123, .173] |
|  | p-value |  | < .001* |  | < .001* |  | < .001* |
| First-person singular | rho |  | .033 |  | -.050 |  | .011 |
|  | 95% CI |  | [-.024, .094] |  | [-.111, .011] |  | [-.016, .040] |
|  | p-value |  | 1 |  | .179 |  | .404 |

| *Bonferroni correction was applied for multiple testing; *significant at alpha < .01; Rho refers to Spearman’s rho correlation coefficient.*  *^a^ using only posts written by victims of DV*  *^b^ using all posts from all subreddits* |
| --- |
